# Multicenter clinical outcomes of hole implantable collamer lens implantation in middle-aged patients

**DOI:** 10.1038/s41598-022-08298-7

**Published:** 2022-03-10

**Authors:** Akihito Igarashi, Kazutaka Kamiya, Kazuo Ichikawa, Yoshihiro Kitazawa, Takashi Kojima, Tomoaki Nakamura, Kimiya Shimizu

**Affiliations:** 1 Department of Ophthalmology, Sanno Hospital, 8-10-16 Akasaka, Minato-ku, Tokyo, 107-0052 Japan; 2 Visual Physiology, School of Allied Health Sciences, Kitasato University, Kanagawa, Japan; 3 Chukyo Eye Clinic, Aichi, Nagoya Japan; 4 Sapia-Tower Eye Clinic, Tokyo, Japan; 5 Department of Ophthalmology, Keio University School of Medicine, Tokyo, Japan; 6 Nagoya Eye Clinic, Aichi, Nagoya Japan

**Keywords:** Health care, Quality of life, Medical research, Outcomes research

## Abstract

To assess the multicenter clinical outcomes of the implantation of hole implantable collamer lens (Hole ICL, ICL KS-AquaPORTTM; STAAR Surgical, Nidau, Switzerland) in patients of 45 years or more. We retrospectively assessed the surgery’s safety, efficacy, predictability, stability, and adverse events before surgery and after the surgery at 1 week; 1, 3, and 6 months; and 1 year, followed by once every year for approximately 2.2 years. A total of 118 eyes of 65 patients aged 45–65 years with myopic refractive errors ranging from − 2.13 to − 18.75 diopters (D) underwent hole ICL implantation and routine postoperative examinations. The average observation period was 2.2 ± 1.0 years. The safety and efficacy indices were 1.08 ±  0.21 and 0.87 ± 0.25, respectively. Manifest refraction changes of − 0.20 ± 0.43 D occurred from 1 month to the final visit after ICL implantation. Eight eyes (6.8%) developed asymptomatic anterior subcapsular cataract (ASC) immediately after surgery, and three eyes (2.5%) developed clinically significant symptomatic nuclear cataracts during the follow-up period. According to our experience, hole ICL implantation offered favorable outcomes in all measures of safety, efficacy, predictability, and stability, even in middle-aged patients, during the 2.2-year observation period.

## Introduction

The posterior chamber phakic intraocular implantable collamer lens (ICL) with a central port (Hole ICL, ICL KS-AquaPORT, STAAR Surgical, Nidau, Switzerland) was first implanted by Kimiya Shimizu in 2007^1^, and the number of ICL surgeries has significantly increased since then due to improved safety. The conventional ICL (before V4 models) had two major problems: (1) Iridectomy was required because the ICL interfered with the intraocular aqueous circulation, and if the iridectomy was incomplete, pupillary block occurred in rare cases^2^. (2) Risk of metabolic cataract progression due to poor circulation of aqueous humor, particularly in cases with low vault (< 230 μm)^3^, high levels of myopia^4^, and an advanced age (over 40 years old)^4^.

When compared to conventional ICLs (V4 and earlier models), hole ICL has a 0.36-mm hole in the center of the lens, which is designed to allow the aqueous humor in the eye to circulate naturally. Therefore, the abovementioned complications of conventional ICL have significantly improved^5^. Due to the risk of cataracts and presbyopia in conventional ICL, the surgical indication for ICL in Japan is 21–45 years of age. However, there are patients aged over 45 years who wish to undergo ICL surgery. Since the extended depth of focus ICL (EVO Viva, STAAR Surgical, Nidau, Switzerland) also obtained the CE mark in July 2020, it is expected that the number of patients aged ≥ 45 years will be increasing further in the future. Therefore, in this multicenter study, we evaluated the safety and efficacy of the clinical outcomes after hole ICL implantation in patients aged ≥ 45 years in Japan.

## Results

### Study population

Preoperative patient demographics are summarized in Table 1. All patients were followed up for more than 1 year, and the observation period was 2.2 ± 1.0 years (range 1–3 years).

**Table 1:** Preoperative demographics of the study population.

Characteristic	Mean ± SD
Observation period (years)	2.2 ± 1.0 years (range 1 to 3 years)
Age (years)	48.9 ± 4.2 years (range 45 to 65 years)
Gender (% female)	47.5%
Manifest spherical equivalent (D)	− 9.15 ± 3.59 D (range − 2.13 to − 18.75 D)
Manifest cylinder (D)	1.11 ± 1.25 D (range 0.00 to 6.00 D)
Log MAR UDVA	1.41 ± 0.26 (range 0.52 to 2.00 )
Log MAR CDVA	− 0.15 ± 0.10 (range − 0.30 to 0.22 )
White-to-white distance (mm)	11.7 ± 0.4 mm (range 10.9 to 12.8 mm)
Anterior chamber depth (mm)	2.99 ± 0.27 mm (range 2.51 to 4.19 mm)
Mean keratometric readings (D)	43.9 ± 1.4 D (range 39.6 to 47.1 D)
Central cornea thickness (μm)	541 ± 39 μm (range 452 to 651 μm)
Intraocular pressure (mmHg)	13.6 ± 2.5 mmHg (range 8 to 21 mmHg)
Endothelial cell density (cells/mm^2^)	2752 ± 263 cells/mm^2^ (range 2153 to 3401 cells/mm^2^)

*Log MAR* logarithm of the minimal angle of resolution, *UDVA* uncorrected distance visual acuity, *CDVA* corrected distance visual acuity.

### Safety outcomes

The log MAR CDVA at 1 week, 1 month, 3 months, 1 year, and the final visit (average: 2.2 years) after surgery was − 0.18 ± 0.08, − 0.18 ± 0.09, − 0.18 ± 0.10, − 0.017 ± 0.09, and 0.17 ± 0.09, respectively. The safety index (mean postoperative CDVA/ mean preoperative CDVA) at 1 week, 1 month, 3 months, 1 year, and the final visit after surgery was 1.10 ± 0.24, 1.10 ± 0.23, 1.11 ± 0.22, 1.08 ± 0.22, and 1.08 ± 0.21, respectively. At the final visit, 62 eyes (52.5%) showed no change in CDVA, 38 eyes (32.2%) gained one line, 1 eye (0.8%) gained two lines, 17 eyes (14.4%) lost one line, and no eye (0%) lost two or more lines.

### Effectiveness outcomes

To evaluate the efficacy of the procedure, we evaluated eyes in which the target refraction was emmetropia (N = 73 eyes). The log MAR UDVA at 1 week, 1 month, 3 months, 1 year, and the final visit (average: 2.2 years) after surgery was − 0.10 ± 0.18, − 0.10 ± 0.17, − 0.10 ± 0.17, − 0.09 ± 0.16, and − 0.07 ± 0.17, respectively. The efficacy index (mean postoperative UDVA/ mean preoperative CDVA) was 0.95 ± 0.29, 0.94 ± 0.28, 0.93 ± 0.28, 0.91 ± 0.26, and 0.87 ± 0.25 at 1 week, 1 month, 3 months, 1 year, and the final visit after surgery, respectively. At 1 week, 1 month, 3 months, 1 year, and the final visit after surgery, 96%, 97%, 96%, 96%, and 96% of eyes, respectively, had UDVAs of 20/40 or better, and 90%, 88%, 90%, 89%, and 86% of eyes, respectively, had UDVAs of 20/20 or better.

### Predictability

**Figure 1:**
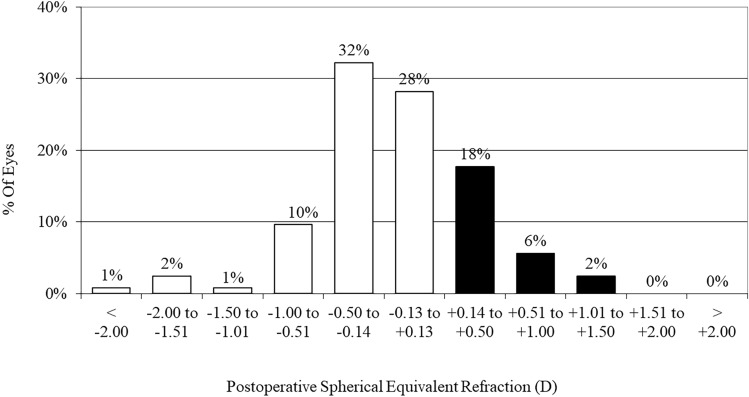
Percentages of eyes within ± 0.5 and ± 1.0 D of the attempted correction (spherical equivalent) after hole implantable collamer lens (ICL) implantation.

At 1 week, 1 month, 3 months, 1 year, and the final visit (average: 2.2 years) after surgery, 80%, 82%, 83%, 80%, and 78% of eyes, respectively, were within ± 0.5 D, and 94%, 96%, 93%, 97%, and 93% of eyes, respectively, were within ± 1.0 D of the attempted correction (Fig. 1).

### Stability

At 1 week, 1 month, 3 months, 1 year, and the final visit (average: 2.2 years) after surgery, the mean manifest spherical equivalent was − 0.35 ± 0.69, − 0.40 ± 0.65, − 0.37 ± 0.68, − 0.48 ± 0.68, and − 0.61 ± 0.71 D, respectively (ANOVA, p = 0.03). Multiple comparisons revealed significant differences between measurements made at 1 week after surgery and at the final visit after surgery (Tukey–Kramer test, p = 0.03). The changes in manifest refraction from 1 month to 1 year and from 1 month to the final visit were − 0.06 ± 0.31 and − 0.20 ± 0.43 D, respectively.

### Intraocular pressure

The IOP showed no significant change from 13.6 ± 2.5 mmHg before the surgery to 14.1 ± 3.2, 13.4 ± 2.4, 13.4 ± 2.6, 13.9 ± 2.8, and 13.9 ± 3.1 mmHg at 1 week, 1 month, 3 months, 1 year, and the final visit (average: 2.2 years) after surgery, respectively (ANOVA, p = 0.21). No significant increase in IOP occurred in any case during the observation period.

### Endothelial cell density

The endothelial cell density significantly decreased from 2752 ± 263cells/mm^2^ preoperatively to 2700 ± 260 cells/mm^2^ at the final visit (average: 2.2 years) postoperatively (Paired t-test, p = 0.01). The mean percentage of endothelial cell loss was1.6 ± 8.1% (range from − 28.0 to + 25.6%) at the final visit (2.2 years postoperatively).

### Secondary surgeries/adverse events

**Figure 2:**
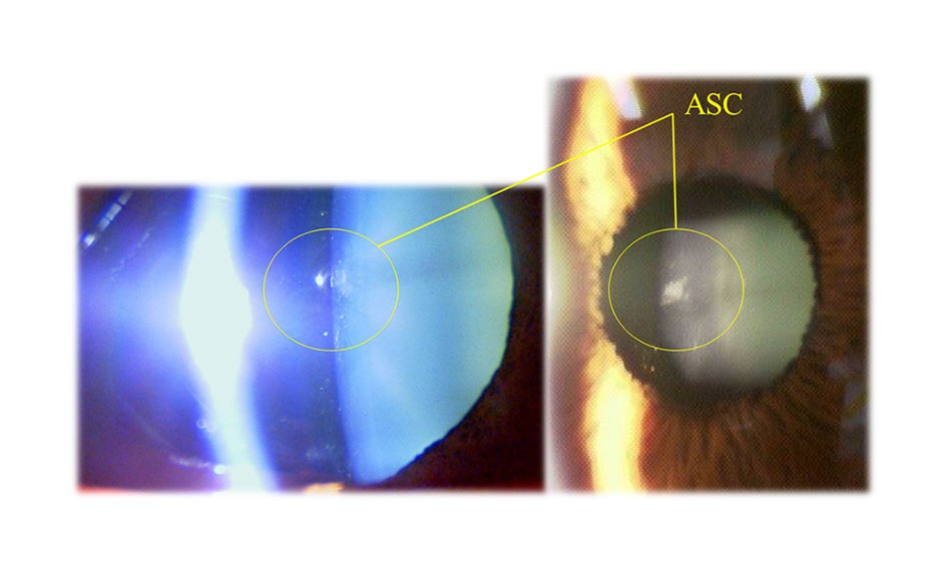
Asymptomatic anterior subcapsular cataract (ASC) cases after hole implantable collamer lens (ICL) implantation.

**Figure 3:**
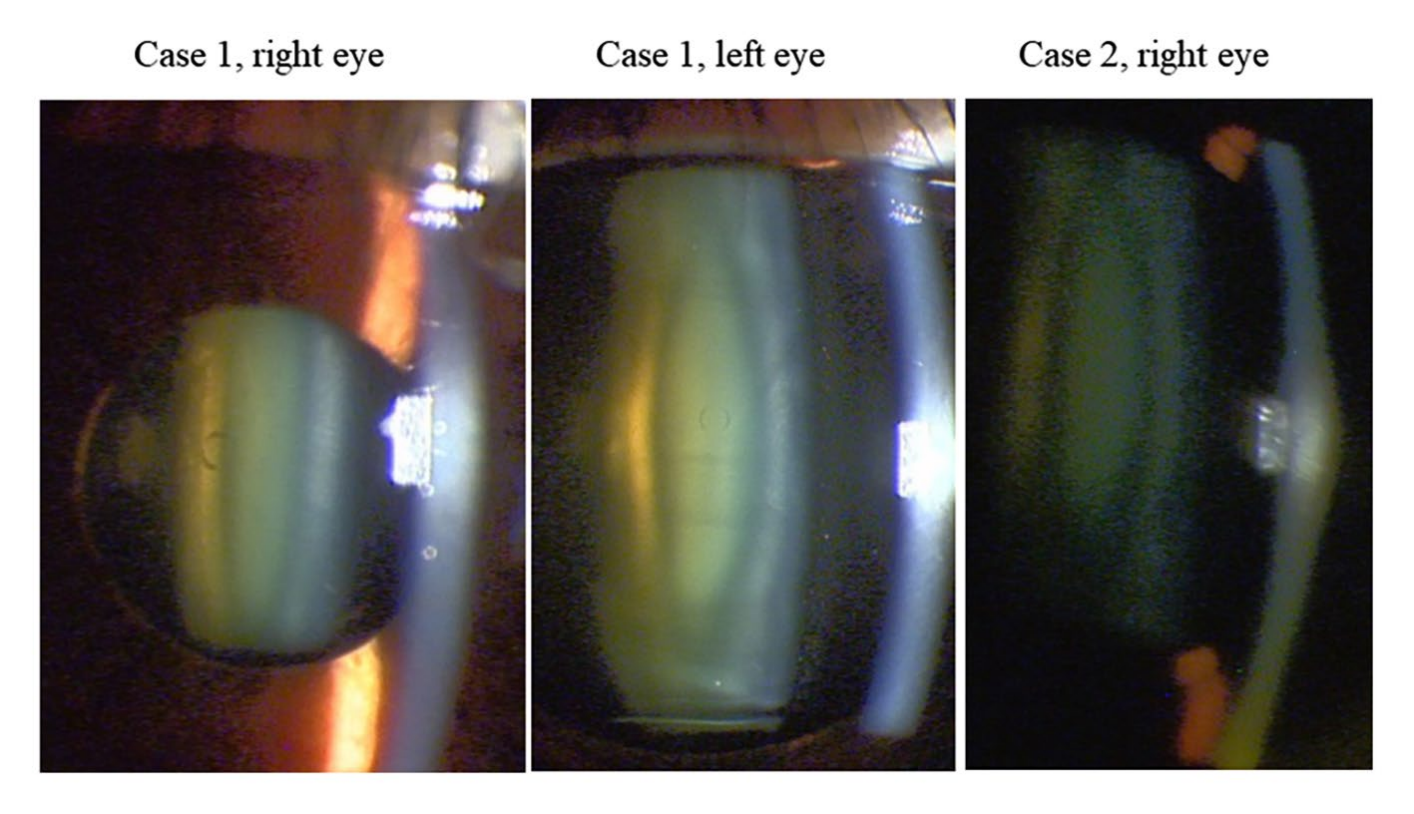
Nuclear cataract cases after hole implantable collamer lens (ICL) implantation.

There were no intraoperative complications, and all implantations were uneventful. Of the 118 eyes examined, 8 eyes (6.8%) developed asymptomatic anterior subcapsular cataract (ASC), but all eyes had a CDVA of 20/20 or better (Table 2). In most cases, ASCs occurred immediately after surgery. Case 1 developed ASC immediately after ICL exchange due to an excessive high vault. ASC occurred under the hole position of the ICL (Fig. 2), possibly due to the excessive suction of the ophthalmic viscosurgical device between the ICL and the crystalline lens through this hole using an irrigation and aspiration tip. The ASC may have been caused due to the contact between the ICL and the anterior surface of the lens during irrigation and aspiration. However, the ASC did not progress thereafter. Only three eyes (2.5%) developed clinically significant nuclear cataracts postoperatively, and these patients complained of poor visibility (double vision and myopic change) (Fig. 3). Simultaneous lens extraction and phacoemulsification with intraocular lens (IOL) implantation were successfully performed in these three eyes, and the patients had improved vision and no other complaints after the surgery. All eyes had high myopia (long axial length) (Table 3). In Table 3, Case 1 underwent binocular cataract surgery with a difference of a few years. The left eye in Case 2 was not indicated for ICL implantation because of a previous history of optic neuritis and poor corrected visual acuity. Subsequently, Case 2 underwent cataract surgery in both eyes at the same time. Of the 46 cases in which toric ICL was used, deteriorating UDVA, changing refraction, or an axis rotation of 10° or more occurred in two eyes (4.3%) postoperatively, which required ICL repositioning. Three eyes (2.5%) underwent ICL exchange due to excessive high vault. No pigment dispersion glaucoma, pupillary block, or any other vision-threatening complications were seen throughout the follow-up period. Table 4 shows the clinical outcomes based on each target refractive power. The results were divided into three target refractive power categories, with no significant differences in the safety and the predictability metrics.

**Table 2:** Asymptomatic anterior subcapsular cataracts (ASC) cases after Hole implantable collamer lens (ICL) implantation.

	Case 1		Case 2	Case 3	Case 4	Case 5		Case 6
	Right eye	Left eye	Left eye	Left eye	Right eye	Right eye	Left eye	Right eye
Age	50	50	59	54	46	53	53	47
Gender	Female	Female	Female	Female	Female	Male	Male	Male
Time of onset	Immediately after surgery (ICL exchange)	Immediately after surgery (ICL exchange)	Immediately after surgery	Immediately after surgery	Immediately after surgery	Immediately after surgery	Immediately after surgery	Post-Op 6 months
Pre. SE (D)	− 6.1	− 6.5	− 18.3	− 11.6	− 14.8	− 16.0	− 12.5	− 13.3
Pre. CDVA	30/20	30/20	20/20	12/20	40/20	24/20	20/20	24/20
CDVA (Final Visit)	20/20 (2 years)	20/13 (2 years)	20/16 (1 year)	20/25 (1 year)	20/13 (3 years)	20/16 (2 years)	20/16 (2 years)	20/13 (3 years)
Vault (CT)	0.6	0.4	0.6	0.7	1.0	0.5	0.3	0.6

*SE* spherical equivalent, *CDVA* corrected distance visual acuity, *CT* corneal thickness.

**Table 3:** The cases of ICL extraction and cataract surgery after Hole implantable collamer lens (ICL) implantation.

	Case 1		Case 2
	Right eye	Left eye	Right eye
Age	46	46	52
Gender	Male	Male	Female
Pre. CDVA	40/20	30/20	24/20
Pre. SE (D)	− 12.3	− 13.0	− 11.8
Pre. vault (CT)	0.7	1.3	1.1
Pre. axial length (mm)	30.1	30.2	27.5
Time to cataract surgery	4.3 years	2.1 years	2.9 years
CDVA (final visit)	24/20	20/20	20/20
SE (D)	− 0.25D	− 2.50D	− 1.75D
Types of cataracts	Nuclear	Nuclear	Nuclear

*CDVA* corrected distance visual acuity, *SE* spherical equivalent, *CT* corneal thickness.

**Table 4:** Clinical outcomes by target refractive power.

	Pre-UDVA	Pre-CDVA	Pre-SE (D)	Target ref (D)	Post-UDVA	Safety index	Efficacy index	Post-SE (D)	Predictability (± 1.0D)
Bilateral Emmetropia (N = 54eyes)	1.38 ± 0.27	− 0.15 ± 0.11	− 9.14 ± 3.70	− 0.10 ± 0.19	− 0.07 ± 0.18	1.11 ± 0.22	0.88 ± 0.26	− 0.40 ± 0.57	93%
Monovision (N = 26eyes DE:13/NDE:13)	DE 1.37 ± 0.22 NDE: 1.44 ± 0.24	DE: − 0.16 ± 0.06 NDE: − 0.16 ± 0.07	DE: − 8.05 ± 3.65 NDE: − 8.55 ± 3.84	DE: − 0.31 ± 0.24 NDE: − 1.14 ± 0.41	DE: − 0.03 ± 0.21 NDE: 0.21 ± 0.32	DE: 1.09 ± 0.17 NDE: 1.06 ± 0.18	DE: 0.80 ± 0.24 NDE: 0.52 ± 0.30	DE: − 0.37 ± 0.42 NDE: − 1.10 ± 0.73	DE: 100% NDE: 100%
Bilateral Intentional-Undercorrection (N = 26eyes)	1.46 ± 0.20	− 0.16 ± 0.09	− 10.17 ± 2.94	− 0.78 ± 0.33	0.08 ± 0.22	1.02 ± 0.20	0.65 ± 0.29	− 0.80 ± 0.63	92%

*UDVA* uncorrected distance visual acuity, *CDVA* corrected distance visual acuity, *SE* spherical equivalent, *DE* dominant eye, *NDE* non-dominant eye.

## Discussion

In this multicenter study, we confirmed excellent results in all measures of safety, efficacy, predictability, and stability of hole ICL implantation in patients older than 45 years throughout the follow-up period of 1 year or more.

Conventional ICLs (before V4 models) have been reported to have good long-term clinical outcomes^9–12^. However, conventional ICL has two drawbacks. One is the need for iridectomy, and the other is the progression of ASC after ICL implantation. Inappropriate iridectomy rarely resulted in pupillary block^2^. The incidence of ASC ranged from 1.1 to 5.9%^5^, and the probability of requiring cataract surgery for vision loss ranged from 0 to 1.8%^5^. The risk of cataract progression was particularly high in patients with an advanced age (over 40 years old)^4^, high myopia (less than − 12.0 D)^4^, and low vault (< 230 μm)^3^. However, with the advent of the hole ICL, these risks have been greatly reduced and safety has been improved^13^.

A meta-analysis of hole ICL^13^ reported a safety index of 1.15 (range: 1.01–1.42, average follow-up period: 13.2 months) and that only 0.2% of eyes lost two or more lines of CDVA, while 95.5% maintained or gained lines of CDVA; the change in CDVA before and after surgery was also good. To date, only one case of pupillary block has been reported after hole ICL implantation^14^, but this case was unique in that the central port of the ICL was blocked with viscoelastic and inflammatory debris. Visually significant cataract has not been reported in patients implanted with hole ICL^13^. The incidence of asymptomatic ASC opacities was 0.49%, but none of the reports had any cases of ICL removal and cataract surgery because of vision loss^13^.

In this multicenter study, the patients were aged 45 years or older and were at a high risk of developing cataract with conventional ICL^4^. There are only a few reports^6–8^ on the postoperative outcomes after hole ICL implantation in middle-aged and older patients, which are summarized in Table 5. All previous reports^6–8^ had short observation periods and small sample sizes. In this study, the mean observation period was 2.2 years (cases with more than 1 year of follow-up), and the safety index was 1.08. None of the eyes lost two or more lines of CDVA, and 85.6% maintained or gained lines of CDVA. This multicenter study demonstrated a 6.8% (8/118) incidence of secondary surgical intervention with an average follow-up period of 2.2 years. Two eyes required rotation of a toric ICL, and three eyes exchanged the ICL due to excessive high vault (> 1000 μm). Three eyes (2.5%) developed clinically significant nuclear cataracts postoperatively, and simultaneous lens extraction and phacoemulsification with IOL implantation were successfully performed. These three eyes also had preoperative mild cataracts of G1.5 to G2 (Emery–Little classification). All cases of nuclear cataract had CDVA > 20/20, but cataract surgery was performed due to decreased UDVA. The prediction of IOL power for cataract surgery in ICL implanted eyes is good^15^, and the patients are also satisfied with their vision. All patients with nuclear cataracts in this study had high myopia with an axial length of ≥ 27.5 mm, which may have influenced the results^16^.

**Table 5:** The reports on Hole implantable collamer lens (ICL) implantation in patients over 40 years of age.

Author	Observation period (number of cases)	Age (min, max)	UDVA (log MAR)	CDVA (log MAR)	± 1.0D (% of eyes)	Post.op SE (D)	ECD (cells/mm^2^) (% of loss)	Cataract
Tañá-Rivero et al.^6^	1 year (33 eyes)	43.5 ± 4.5 (40, 56)	0.88 ± 0.16 (decimal)	0.96 ± 0.09 (decimal)	93.9	− 0.09 ± 0.47	2516 ± 234 (2.04)	0%
Kamiya et al.^7^	6 months (34 eyes)	46.1 ± 4.2 (40, 53)	− 0.04 ± 0.18	− 0.19 ± 0.09	100	DE: − 0.08 ± 0.17 NDE: − 0.65 ± 0.29	–	0%
Takahashi et al.^8^	6 months (42 eyes)	45.0 ± 3.8 (40, 53)	− 0.03 ± 0.20	− 0.19 ± 0.08	100	–	–	0%
Current study	2.2 years (118 eyes)	48.9 ± 4.2 (45, 65)	− 0.07 ± 0.17	− 0.17 ± 0.09	93	− 0.61 ± 0.71	2700 ± 260 (1.6)	6.8%: Asymptomatic ASC cases 2.5%: Symptomatic NC cases

*UDVA* uncorrected distance visual acuity, *CDVA* corrected distance visual acuity, *SE* spherical equivalent, endothelial cell density, *DE* dominant eye, *NDE* non-dominant eye, *ASC* anterior subcapsular cataract, *NC* nuclear cataract, *Log MAR* logarithm of the minimal angle of resolution.

Asymptomatic ASC opacities occurred in 6.8% (8/118) of cases. This is a high rate compared to previous reports^17–19^, but seven eyes developed ASC opacities immediately after surgery. In these cases, the ASC was localized just below the central port of the lens, suggesting that it was caused by an intraoperative irrigation technique. The suction hole was vigorously directed toward the central hole during irrigation and aspiration, and we thought that the ASC was caused by the contact of the anterior surface of the lens with the posterior surface of the ICL during aspiration. Steinwender et al.^20^ reported similar cases and noted that after changing the surgical technique to a very gentle irrigation and keeping the cannula near the main incision, more than 90 phakic IOL implantations were performed in the clinic during a follow-up period of 14 months, with no further occurrence of ASC. This may imply that ASC can easily occur by mechanical contact with the lens in older patients, since no ASCs have been observed in young patients using this technique. In this study, patients with asymptomatic ASC opacities did not have ASC progression or CDVA decline and did not require additional surgery.

As a limitation to this study, we believe that a longer follow-up is necessary. In addition, we could not measure the objective postoperative vault because not all hospitals had anterior segment optical coherence tomography. Further, we included some cases with shallow anterior chamber depth (> 2.5 mm) based on previous reports^21^. In the future, we believe that a more detailed investigation of the relationship between ICL size and postoperative complications is desirable.

In conclusion, this multicenter study supports the view that hole ICL implantation offered good results in all measures of safety, efficacy, predictability, and stability in patients over 45 years old during the 2.2-year observation period. However, we must be careful of mechanical lens contact associated with intraoperative manipulation in older patients. In addition, since nuclear cataracts may occur with aging in patients with severe myopia, prior explanation is important. The number of ICL surgeries in middle-aged and elderly patients is expected to increase in the future, and we hope that the results of this study will be of help to these patients.

## Methods

This was a multicenter study performed at Kitasato University, Nagoya Eye Clinic, Sapia Tower Eye Clinic, and Sanno Hospital in Japan. A total of 118 eyes of 65 consecutive patients (males: 62eyes and females: 56 eyes) who underwent implantation of hole ICL for the correction of myopia and myopic astigmatism and regularly returned for postoperative examination were included in this retrospective observational study. In this study, 106 eyes of 53 patients were operated on both eyes, and 12 eyes were operated on only one eye. The inclusion criteria for this surgical technique were as follows: unsatisfactory correction with spectacles or contact lenses, ≥ 45 years of age, stable refraction for at least 6 months, myopia ranging from − 2.0 to − 20.0 diopters (D), anterior chamber depth ≥ 2.5 mm, endothelial cell density ≥ 2000 cells/mm^2^, no history of ocular surgery, progressive corneal degeneration, cataract, glaucoma or uveitis, and follow-up of ≥ 1 year. Eyes with keratoconus were excluded from the study by using the keratoconus screening test of Placido disk videokeratography (TMS-2, Tomey, Nagoya, Japan). Before surgery, the horizontal white-to-white distance and anterior chamber depth (from the corneal endothelium to the anterior surface of the lens) were measured using a scanning-slit topograph (Orbscan IIz, Bausch & Lomb, Rochester, US), and the axial length was measured using partial coherence laser interferometry (IOL Master; Carl Zeiss AG, Oberkochen, Germany). Before surgery and 1 day; 1 week; 1, 3, and 6 months; and 1, 2, and 3 years after surgery, we determined the following: logarithm of the minimal angle of resolution (log MAR) of uncorrected distance visual acuity (UDVA), log MAR of corrected distance visual acuity (CDVA), manifest refraction (spherical equivalent), intraocular pressure (IOP), endothelial cell density, mean keratometric reading, and axial length, in addition to the usual slit-lamp biomicroscopic and fundoscopic examinations. The size of ICL was selected based on the nomogram provided by the manufacturer (STAAR Surgical).

The study was approved by the Institutional Review Board of International University of Health and Welfare (21-S-10) and followed the tenets of the Declaration of Helsinki. Written informed consent was obtained from all patients after explanation of the nature and possible consequences of the study.

### Implantable collamer lens power calculation

ICL power calculation was performed by the manufacturer (STAAR Surgical) using a modified vertex formula. In this study, we set the target refractive power taking presbyopia into account, as described previously^6–8^. The target refractive power was emmetropia in 50.9% (54/106), monovision in 24.5% (26/106), and intentional under-correction in 24.5% (26/106). The target refractive power was individually determined by contact lens simulation and the patient's lifestyle. These groupings by the target refractive power were individually performed by each case in which both eyes were operated.

### Implantable collamer lens surgical procedure

All ICL implantation surgeries were performed using a standardized method in all surgical centers. After topical anesthesia, ICL was inserted through a 3-mm clear corneal incision using an injector cartridge (STAAR Surgical) after placement of a viscosurgical device (Opegan; Santen, Osaka, Japan) into the anterior chamber. The ICL was placed in the posterior chamber, the viscosurgical device was completely washed out of the anterior chamber with a balanced salt solution, and a myotic agent was instilled. All surgeries were uneventful, and no intraoperative complication was observed. After surgery, steroids (0.1% betamethasone; Rinderon; Shionogi, Osaka, Japan) and antibiotics (0.3% levofloxacin; Cravit; Santen, Osaka, Japan) were topically administered four times daily for two weeks, and the dose was gradually reduced thereafter.

### Statistical analysis

All statistical analyses were performed using StatView version 5.0 (SAS, Cary, NC, USA). One-way analysis of variance (ANOVA) was used to analyze the time course of changes, along with the Tukey–Kramer test for multiple comparisons. The paired t-test was used to compare the pre- and post-surgical data. The Pearson correlation coefficient was used to assess the correlation between the changes in manifest spherical equivalent and axial length. The results are expressed as mean ± standard deviation, and a value of p < 0.05 was considered statistically significant.
